# Fractional dynamic system simulating the growth of microbe

**DOI:** 10.1186/s13662-021-03498-3

**Published:** 2021-07-29

**Authors:** Samir B. Hadid, Rabha W. Ibrahim

**Affiliations:** 1 Nonlinear Dynamics Research Center (NDRC), Ajman University, Ajman, UAE; 2 Department of Mathematics and Sciences, College of Humanities and Sciences, Ajman University, 346 Ajman, UAE; 3 IEEE, 94086547, Kuala Lumpur, 59200 Malaysia

**Keywords:** Fractional calculus, Fractional differential operator, Fractional equation

## Abstract

There are different approaches that indicate the dynamic of the growth of microbe. In this research, we simulate the growth by utilizing the concept of fractional calculus. We investigate a fractional system of integro-differential equations, which covers the subtleties of the diffusion between infected and asymptomatic cases. The suggested system is applicable to distinguish the presentation of growth level of the infection and to approve if its mechanism is positively active. An optimal solution under simulation mapping assets is considered. The estimated numerical solution is indicated by employing the fractional Tutte polynomials. Our methodology is based on the Atangana–Baleanu calculus (ABC). We assess the recommended system by utilizing real data.

## Introduction

Integro-differential dynamic system of equations simulates various states from science and engineering corresponding to the analysis, control, and optimization studies. The main model in this direction is the Wilson–Cowan system, which designs the dynamics of connections between populations of very inhibitory system in cells or neurons. It was developed by Hugh R. Wilson and Jack D. Cowan [1]. The system and its generalizations have been extensively utilized in forming neuronal or cell populations [2]. The system is significant traditionally because it utilizes phase plane approaches and mathematical solutions to designate the reactions of neuronal populations to motivations. The general system involves simple integro-differential equations, therefore, limit cycle performance (neural fluctuations) and stimulus-dependent suggested reactions are expected. The key results contain the solvability of multiple stable situations and hysteresis in the population’s reaction.

Coronavirus (COVID-19) has been an infectious virus molded by a recently exposed coronavirus. It has been recorded by the World Health Organization (WHO), it is a pandemic. The first WHO warning of dyed-in-the-wool cases of COVID-19 indicated on January 2020 with 282 cases (see [3, 4]). There is an increasing number of research works that develop the growth of the COVID-19 infection by using an ordinary dynamic system [5] and fractal-fractional dynamic system [6]. Utilizing the recent information from European and African countries, Atangana and Araz presented numerous statistical analyses [7, 8]. Musa et al. [9] introduced a nonlinear 4D-system of ordinary differential equations describing COVID-19. Atangana [10] formulated a numerical design using the Newton polynomial. Other strategies can be located in efforts by Memon et al. [11]. Newly, numerous mathematical simulations have been indicated to realize the coronavirus infection. Supreme of these representations are based on classical integer-order derivative or classical fractional differential operators, which cannot get the vanishing memory and boundary performance found in numerous biological phenomena. Consequently, we investigate the coronavirus disease in this study by discovering the dynamics of COVID-19 infection utilizing the fractional Caputo derivative.

The terminal coronavirus continues to blow out across the globe, and mathematical models can be utilized to display suspected, recovered, and deceased coronavirus patients, as well as how many persons have been tested or even vaccinated. Consequently, mathematical and statistical solutions of the infected human beings overall can decrease the risk of future COVID-19 spread. In this study, we aim to generalize the Wilson–Cowan system (WCS) utilizing the concept of fractional calculus to study the growth of COVID-19 population. This investigation includes a dynamic term, which is the exponential law to discover and realize the graph of the growth. The solvability of the system is indicated by using the optimal point theorem of simulation function. Other behaviors are indicated such as the approximated solvability using the fractional Tutte polynomials.

## Preparations

This section deals with some concepts and the properties of these concepts.

### ABC-definition

In recent decades, numerous physical issues have been exposed using the fractional calculus. The essential explanations for employing fractional calculus are that various measures, structures, and inequities display capability to remember the past or nonlocal possessions. The basic outlook and appearances of fractional calculus and fractional differential equations are recognized in various reviews. Most researches focus on the derivatives, which include kernels. For instance, the main difference between the Caputo operator, the Caputo–Fabrizio operator [12], and others is that the Caputo operator is communicated by giving a power law, the Caputo–Fabrizio operator is adapted by utilizing an exponential growth act. The Atangana–Baleanu operator is presented by signifying the extended Mittag-Leffler function [13].

#### Definition 2.1

Let $\Lambda ^{\mu }$, $\mu \in (0,1)$ be the Atangana–Baleanu operator of order *μ* of a function *χ* fulfilling

$$ \Lambda ^{\mu } \chi (t)= \frac{B(\mu )}{1-\mu } \int _{0}^{t} \chi '( \tau ) \Xi _{\mu } \biggl(\frac{-\mu }{1-\mu } (t-\tau )^{\mu } \biggr)\,d\tau ,\quad t\in [0,\infty ), $$

where $B(\mu )$ indicates a normalization function, Ξ represents the Mittag-Leffler function. Corresponding to $\Lambda ^{\mu }$, the ABC integral is realized by

$$ \jmath ^{\mu }\chi (t)= \frac{(1-\mu ) }{B(\mu )}\chi (t)+ \frac{\mu }{B(\mu )\Gamma (\mu )} \int _{0}^{t}\chi (\tau ) (t-\tau )^{ \mu -1}\,d\tau . $$

#### Example 2.1

Consider the function $\chi (t)=t^{m}$, then the ABC integral becomes

$$ \jmath ^{\mu }t^{m}= \frac{(1-\mu ) }{B(\mu )}t^{m}+ \frac{\mu \Gamma (m+1)}{B(\mu )\Gamma (m+1+ \mu )}t^{m+\mu }. $$

In our study, since we focus on the approximated solutions, we assume that $B(\mu ) \rightarrow 1$ for all $\mu \in (0,1)$. Applications of this calculus in COVID-19 can be located in [14–16].

### Approximate point theorem

We deal with the resulting notion of cyclic ∁-condensing operator. Let ð be a measure of noncompactness on a Banach space $\mathbb{X}$ and *A*, *B* be nonempty and convex subsets of $\mathbb{X}$ (see [17]).

#### Definition 2.2

A function $f: \mathbb{R}_{+} \times \mathbb{R}_{+} \rightarrow \mathbb{R}$ is called simulation if and only if $f(0,0)=0$, $f(x_{1},x_{2})< x_{2}-x_{1}$, where $x_{2},x_{1}>0$ and $\lim_{i \rightarrow \infty } \sup f(x_{i},y_{i})<0$ for $x_{i}< y_{i}$ and $\lim_{i \rightarrow \infty } x_{i}=\lim_{i \rightarrow \infty } y_{i}=0$.

#### Definition 2.3

Let $Y: A\cup B \rightarrow A\cup B$ be an operator. It is *f*-cyclic condensing (CC) if, for every nonempty, bounded, convex, and closed subset $(A_{1},B_{1})$,

$$ f \bigl[\eth \bigl( Y(A_{1})\cup Y(B_{1})\bigr] \bigr) , \eth ( A_{1} \cup B_{1} )] \geq 0, \quad (A_{1},B_{1}) \subset (A,B). $$

#### Lemma 2.2

*A relatively nonexpansive cyclic* *f*-*condensing mapping* $Y: A\cup B \rightarrow A\cup B$ *recognizes a best proximity point*.

## Results

We let $\mathbb{N} (t) $ be the accumulation number of infected people, which represents the sum of the number of the standard infected persons $\chi (t)$ and that of the asymptomatic transmission ones $\Upsilon (t)$: $\mathbb{N} (t) =\chi (t) + \Upsilon (t)$. Take into account that $\chi (t)$ includes people who have previously been diseased. Therefore, there are rate functions joining *χ* and ϒ. The following integro-differential system indicates the generalization of WCS. We suggest the generalization by using the ABC formula of fractional calculus as follows:

1 $$ \begin{aligned} & \Lambda ^{\mu }\chi (\varsigma )= \Psi _{1} \biggl( \varsigma , \chi (\varsigma ), \int _{\tau _{1}}^{\tau _{1}+\tau _{2}} \phi _{1}\bigl(\varsigma , \tau ,\chi (\tau )\bigr)\,d\tau , \int _{\tau _{1}}^{ \varsigma } \psi _{1}\bigl(\varsigma , \tau ,\chi (\tau )\bigr)\,d\tau \biggr), \\ &\chi (\tau _{1})=\chi _{1}, \\ & \Lambda ^{\mu }\Upsilon (\varsigma )= \Psi _{2} \biggl( \varsigma , \Upsilon (\varsigma ), \int _{\tau _{1}}^{\tau _{1}+\tau _{2}} \phi _{2}\bigl( \varsigma , \tau ,\Upsilon (\tau )\bigr)\,d\tau , \int _{\tau _{1}}^{ \varsigma } \psi _{2}\bigl(\varsigma , \tau ,\Upsilon (\tau )\bigr)\,d\tau \biggr), \\ &\Upsilon (\tau _{1})= \Upsilon _{1}, \end{aligned} $$

where the variables are defined in different intervals: $\Pi =[\tau _{1}-\tau _{2},\tau _{1}+\tau _{2}]$, $\Pi _{\chi }= [\chi _{1}- \epsilon ,\chi _{1}+ \epsilon ]$, $\Pi _{\Upsilon }= [\Upsilon _{1}-\epsilon ,\Upsilon _{1}+ \epsilon ]$, and $\Pi _{\epsilon }=[\tau _{1}-\epsilon ,\tau _{1}+\epsilon ]$.

To study the solvability of system (1), we formulate the following assumptions:

1. All the functions are continuous in $\mathbb{R}$ such that $\phi _{1}: \Pi \times \Pi \times \Pi _{\chi }\rightarrow \mathbb{R}$, $\phi _{2}: \Pi \times \Pi \times \Pi _{\Upsilon }\rightarrow \mathbb{R}$, $\Psi _{1}: \Pi _{\epsilon }\times \Pi _{\chi }\times \Pi _{\chi }\times \Pi _{\chi }\rightarrow \mathbb{R}$, $\Psi _{2}: \Pi _{\epsilon }\times \Pi _{\Upsilon }\times \Pi _{\Upsilon }\times \Pi _{\Upsilon }\rightarrow \mathbb{R}$ and *χ*, ϒ are inside the nonempty, bounded, closed, and convex sets $\intercal _{1}\subset C(\Pi _{\epsilon },\mathbb{R})$ and $\intercal _{2} \subset C(\Pi _{\epsilon },\mathbb{R})$ respectively.
2. For a sup.norm, we suppose that $\|\chi _{1}-\Upsilon _{1}\|\leq \epsilon \|\chi -\Upsilon \|$, $0< \epsilon \leq 1$, so that $\operatorname{dis} (\intercal _{1},\intercal _{2})=\|\chi _{1}-\Upsilon _{1}\|$. In addition, for all $\chi \in \intercal _{1}$ and $\Upsilon \in \intercal _{2}$, we suppose that there occurs a positive constant $\rho >0$ fulfilling
  $$ \Vert \Psi _{1}-\Psi _{2} \Vert \leq \rho \bigl( \Vert \chi -\Upsilon \Vert - \Vert \chi _{1}-\Upsilon _{1} \Vert \bigr). $$
3. For any $\Pi _{\chi }$, $\Pi _{\Upsilon }$, there exists a positive function  which is upper semi-continuous and achieves  and

Here, we introduce our theorem for the solvability of system (1). We define an operator $\mathbb{Q}: \intercal _{1} \cup \intercal _{2} \rightarrow C(\Pi _{\epsilon },\mathbb{R})$ as follows:

2 $$ \mathbb{Q}(\varsigma ):= \textstyle\begin{cases} \Upsilon _{1}+ (1-\mu )\Psi _{1}+\frac{\mu }{\Gamma (\mu )} \int _{ \tau _{1}}^{\varsigma }\Psi _{1}(\eta )(\varsigma -\eta )^{\mu -1}\,d\eta & \text{if } \chi \in \intercal _{1}, \\ \chi _{1}+ (1-\mu ) \Psi _{2}+\frac{\mu }{\Gamma (\mu )} \int _{\tau _{2}}^{\varsigma }\Psi _{2}(\eta )(\varsigma -\eta )^{\mu -1}\,d\eta & \text{if } \Upsilon \in \intercal _{2}. \end{cases} $$

### Theorem 3.1

*Consider system* (1) *satisfying hypotheses* (A1)*–*(A3). *Then it has an optimal solution in* $C(\Pi _{\epsilon },\mathbb{R})$, *whenever*

$$\begin{aligned}& \rho < \frac{\Gamma (\mu )}{\Gamma (\mu )+(1-\mu )+\bar{\epsilon }_{\mu }}, \\& ( \rho >0, 0< \mu < 1, \bar{\epsilon }_{\mu }>0 ). \end{aligned}$$

### Proof

Consider the operator $\mathbb{Q}$ and $B(\mu )\rightarrow 1$. We aim to show that $\mathbb{O}$ is a cyclic operator. Let $\chi \in \intercal _{1}$, then we get

$$\begin{aligned} \bigl\Vert (\mathbb{Q}\chi )- \Upsilon _{1} \bigr\Vert &= \biggl\Vert (1-\mu ) \Psi _{1}+ \frac{\mu }{\Gamma (\mu )} \int _{\tau _{1}}^{\varsigma }\Psi _{1}(\eta ) (\varsigma - \eta ) ^{\mu -1}\,d\eta \biggr\Vert \\ &\leq (1-\mu ) \Vert \Psi _{1} \Vert + \frac{\mu }{\Gamma (\mu )} \int _{\tau _{1}}^{\varsigma } \bigl\Vert \Psi _{1}( \eta ) (\varsigma -\eta )^{\mu -1} \bigr\Vert d \eta \\ &\leq (1-\mu ) \Vert \Psi _{1} \Vert + \frac{\mu }{\Gamma (\mu )} \Vert \Psi _{1} \Vert \int _{\tau _{1}}^{\varsigma }(\varsigma -\eta )^{\mu -1}\,d\eta \\ &\leq \Vert \Psi _{1} \Vert \biggl( 1+ \frac{(\varsigma -\tau _{1})^{\mu }}{\Gamma (\mu )} \biggr) \\ &:= \Vert \Psi _{1} \Vert \biggl( 1+ \frac{\epsilon _{1}^{\mu }}{\Gamma (\mu )} \biggr) \\ &:= S_{1} (_{1}\epsilon _{\mu }),\quad {}_{1} \epsilon _{\mu }:= 1+ \frac{\epsilon _{1}^{\mu }}{\Gamma (\mu )}, \end{aligned}$$

where $S_{1}:=\sup (\Psi _{1})=\|\Psi _{1}\|$. By letting $(_{1}\epsilon _{\mu })< \frac{\bar{\epsilon }_{\mu }}{\max \{S_{1},S_{2}\}}$, where $\bar{\epsilon }_{\mu }:=\max \{ _{1}\epsilon _{\mu }, _{2}\epsilon _{\mu }\}$ and $S_{2}:=\sup (\Psi _{2})$, we have

$$ \bigl\Vert (\mathbb{Q}\chi )-\Upsilon _{1} \bigr\Vert < \bar{ \epsilon }_{\mu }, \quad \forall \chi \in \intercal _{1}. $$

Thus, $\mathbb{Q}\chi \in \intercal _{2}$. In the same manner, we conform that, for $\Upsilon \in \intercal _{2}$, this indicates that

$$ \Vert \mathbb{Q}\Upsilon - \chi _{1} \Vert < \bar{\epsilon }_{\mu }, $$

and hence $\mathbb{Q}y \in \intercal _{1} $. We conclude that $\mathbb{Q}$ is cyclic. The above conclusion shows that the set $\mathbb{Q}(\intercal _{1})$ is bounded in ⊺_2_, and the set $\mathbb{Q}(\intercal _{2})$ is bounded in ⊺_1_.

Recall that $\phi \in \intercal _{1} \cup \intercal _{2}$ indicates an optimum outcome of system (1) if and only if $\operatorname{dist} (\intercal _{1} \cup \intercal _{2})=\|\phi -\mathbb{Q}\phi \|$. Therefore, we have to prove this fact. Next, we aim to prove that $\mathbb{Q}(\intercal _{1} )$ is equicontinuous in ⊺_2_. For *ς* and $\varsigma '$, we have

$$\begin{aligned} & \bigl\Vert \mathbb{Q}\chi (\varsigma )- \mathbb{O} \chi \bigl( \varsigma '\bigr) \bigr\Vert \\ &\quad =\frac{\mu }{\Gamma (\mu )} \biggl\Vert \int _{\tau _{1}}^{\varsigma }\Psi _{1}(\eta ) (\varsigma -\eta )^{\mu -1}\,d\eta - \int _{ \tau _{1}}^{\varsigma '} \Psi _{1}(\eta ) \bigl( \varsigma ' -\eta \bigr)^{\mu -1}\,d\eta \biggr\Vert \\ &\quad \leq \frac{\mu }{\Gamma (\mu )} \biggl\vert \int _{\varsigma }^{\varsigma ' } \bigl\Vert \Psi _{1}( \eta ) \bigr\Vert \bigl(\varsigma -\varsigma ' \bigr)^{\mu -1}\,d\eta \biggr\vert \\ &\quad \leq \frac{1}{\Gamma (\mu )}S_{1} \bigl\vert \varsigma - \varsigma ' \bigr\vert ^{\mu } \\ &\quad \leq \bar{\epsilon }_{\mu }S_{1}, \end{aligned}$$

which implies that $\mathbb{Q}(\intercal _{1}) $ is equicontinuous in ⊺_2_. In a similar manner, we confirm that $\mathbb{Q}(\intercal _{2}) $ is equicontinuous in ⊺_1_. As a consequence and via the Arzela–Ascoli theorem, we point that the pair $(\intercal _{1},\intercal _{2})$ is relatively compact. Now, we have to show that $\mathbb{Q}$ is relatively nonexpansive.

For $(\chi ,\Upsilon ) \in (\intercal _{1},\intercal _{2})$, we inform that

$$\begin{aligned} &\bigl\Vert \mathbb{Q}\chi (\varsigma )- \mathbb{Q} \Upsilon ( \varsigma ) \bigr\Vert \\ &\quad = \biggl\Vert \Upsilon _{1}+ (1- \mu ) \Psi _{1}+ \frac{\mu }{\Gamma (\mu )} \int _{\tau _{1}}^{\varsigma }\Psi _{1}( \varsigma -\eta )^{\mu -1}(\eta )\,d\eta \\ & \qquad {}- \chi _{1}- (1-\mu ) \Psi _{2}-\frac{\mu }{\Gamma (\mu )} \int _{ \tau _{1}}^{\varsigma } \Psi _{2}(\eta ) ( \varsigma -\eta )^{\mu -1}\,d\eta \biggr\Vert \\ &\quad \leq \Vert \chi _{1}-\Upsilon _{1} \Vert + (1-\mu ) \bigl\Vert \Psi _{1}(\varsigma )- \Psi _{2}(\varsigma ) \bigr\Vert \\ &\qquad {} +\frac{\mu }{\Gamma (\mu )} \biggl\vert \int _{ \tau _{1}}^{\varsigma } \bigl\Vert \Upsilon _{1}(\eta )-\Upsilon _{2}(\eta ) \bigr\Vert ( \varsigma - \eta )^{\mu -1}\,d\eta \biggr\vert \\ &\quad \leq \epsilon \Vert \chi -\Upsilon \Vert + (1-\mu ) \rho \bigl( \Vert \chi - \Upsilon \Vert - \Vert \chi _{1}-\Upsilon _{1} \Vert \bigr)+ \frac{\rho \bar{\epsilon }_{\mu }}{\Gamma (\mu )} \bigl( \Vert \chi - \Upsilon \Vert - \Vert \chi _{1}-\Upsilon _{1} \Vert \bigr) \\ &\quad \leq \epsilon \Vert \chi -\Upsilon \Vert + (1-\mu ) \rho \bigl( \Vert \chi - \Upsilon \Vert - \Vert \chi _{1}-\Upsilon _{1} \Vert \bigr)+ \frac{\rho \bar{\epsilon }_{\mu }}{\Gamma (\mu )} \bigl( \Vert \chi - \Upsilon \Vert - \Vert \chi _{1}-\Upsilon _{1} \Vert \bigr) \\ &\quad = \biggl[\epsilon +(1-\mu )\rho + \frac{\rho \bar{\epsilon }_{\mu }}{\Gamma (\mu )}\biggr] \Vert \chi - \Upsilon \Vert -\biggl[(1- \mu )\rho + \frac{\rho \bar{\epsilon }_{\mu }}{\Gamma (\mu )}\biggr] \Vert \chi _{1}- \Upsilon _{1} \Vert . \end{aligned}$$

But *ϵ* is an arbitrary constant, thus when $\epsilon \rightarrow 0$, we have the inequality

$$ \begin{aligned} \bigl\Vert \mathbb{Q}\chi (\varsigma )- \mathbb{Q} \Upsilon ( \varsigma ) \bigr\Vert & \leq \biggl[(1-\mu )\rho + \frac{\rho \bar{\epsilon }_{\mu }}{\Gamma (\mu )}\biggr] \bigl( \Vert \chi - \Upsilon \Vert \bigr) \\ &< \Vert \chi -\Upsilon \Vert . \end{aligned} $$

This indicates that $\mathbb{Q}$ is relatively nonexpansive.

We proceed to show that $\mathbb{Q}$ is *f*-condensing. Assume that $(\Pi _{\chi },\Pi _{\Upsilon })\subseteq (\intercal _{1},\intercal _{2})$ is a nonempty, bounded, closed, and convex set such that
 Thus, we obtain
 By putting , then we arrive at
 Hence, the necessary requirements of Lemma 2.2 are achieved. Thus, the operator $\mathbb{Q}$ has the best proximity point and thus system (1) has an optimal solution.

This completes the proof. □

### Numerical structures

In this subsection, we introduce some numerical systems pointing to utilize Theorem 3.1. The chief assumption in Theorem 3.1 is $\rho < \frac{\Gamma (\mu )}{\Gamma (\mu )+(1-\mu )+\bar{\epsilon }_{\mu }}$. This inequality is very informal to fulfill likening with other existence theorems attaining (A1–A3). Theorem 3.1 designates that the system attractive formula (1) admits an optimal solution. This kind of result is very significant in dynamic and control systems. By this result, one can investigate the stability, oscillatory solution, and other performances of the resolution.

#### Example 3.2

3 $$ \begin{aligned} & \Lambda ^{0.9} \chi ( \varsigma )= \chi ( \upsilon _{1}- \upsilon _{2} \Upsilon ),\qquad \chi (0)=\chi _{0}, \\ & \Lambda ^{0.9} \Upsilon (\varsigma )= \Upsilon ( \upsilon _{3} \chi -\upsilon _{4} ),\qquad \Upsilon (0)=\Upsilon _{0}. \end{aligned} $$

By using Mathematica 11.2, the solution is realized by the integral

$$\begin{aligned}& \int _{\tau _{0}=0}^{\chi [\varsigma ] 1/\tau } \frac{1}{(1 + W (-(\upsilon _{2})/(\upsilon _{1}) \exp ((\upsilon _{3} \tau - c )/\upsilon _{1}) \tau ^{-\upsilon _{4}\upsilon _{1}}) )}\,d\tau \approx \upsilon _{1}\varsigma + c, \\& \Upsilon [\varsigma ] = (-\upsilon _{1}/\upsilon _{2}) \times W \bigl((- \upsilon _{2}/\upsilon _{1}) \exp \bigl( \bigl(\upsilon _{3} \chi [\varsigma ] - c\bigr)/ \upsilon _{1}\bigr) \chi [\varsigma ]^{-\upsilon _{4}/\upsilon _{1}} \bigr), \end{aligned}$$

where *c* is a constant and *W* represents the product log function. As an application of Theorem 3.1, we assume that $(\chi _{0},\Upsilon _{0})=(1,1)$ and $\rho = (\upsilon _{2}\upsilon _{3}-\upsilon _{1}\upsilon _{4})$, where

$$ \rho < \frac{\Gamma (\mu )}{\Gamma (0.9)+(1-0.9)+\bar{\epsilon }_{0.9}}= \frac{1.06}{3.10}=0.341. $$

**Figure 1:**
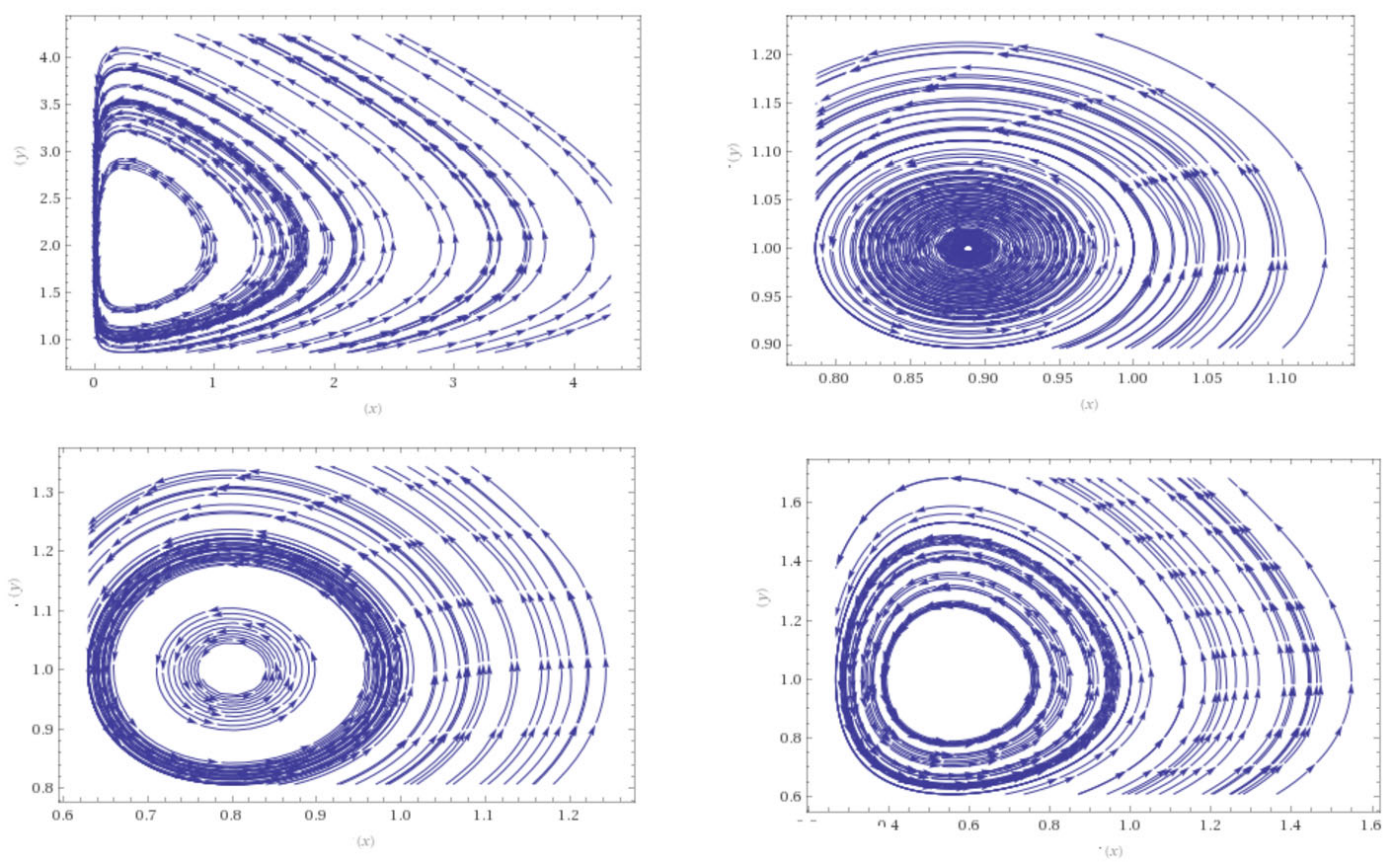
Results of (3) with various values of *ρ*. From the upper left, the solution is for $(\upsilon _{1},\upsilon _{2},\upsilon _{3},\upsilon _{4})=(2,1,0.4,0.1)$, while the lower left is the optimal outcome for $(1,1,0.9,0.8) $. The upper and lower right graphs represent the solutions for $(1,1,1,0.8) $ and $(1,0.9,1,0.8) $ respectively. We indicate that the cyclic result for these values is based on the fact that $\mathbb{Q}$ is cyclic

For instance, $(\upsilon _{1},\upsilon _{2},\upsilon _{3},\upsilon _{4})=(2,1,0.4,0.1)$, we attain $\rho =0.2<0.341$; thus, by Theorem 3.1, system (3) admits an optimal solution converging to a limit cycle. In another case, suppose that $(\upsilon _{1},\upsilon _{2},\upsilon _{3},\upsilon _{4})=(1,1,0.9,0.8)$, then $\rho =0.1<0.341$. This implies that system (3) admits optimal solution converging to a limit cycle. Similarly, for $(\upsilon _{1},\upsilon _{2},\upsilon _{3},\upsilon _{4})=(1,1,1,0.8) \Rightarrow \rho = 0.2<0.341$ and $(\upsilon _{1},\upsilon _{2},\upsilon _{3},\upsilon _{4})=(1,0.9,1,0.8) \Rightarrow \rho = 0.1<0.341$. Figure 1 represents various cases considering the value of *ρ*.

#### Example 3.3

Consider the following system:

4 $$ \begin{aligned} & \Lambda ^{0.5} \chi ( \varsigma )= \Upsilon , \qquad \chi (0)=\chi _{0} \\ &\Lambda ^{0.5}\Upsilon (\varsigma )=-\chi +\rho \Upsilon ,\qquad \Upsilon (0)=\Upsilon _{0}, \end{aligned} $$

where the value of *ρ* achieves

$$ \rho < \frac{\Gamma (0.5)}{\Gamma (0.5)+(1-0.5)+\bar{\epsilon }_{0.5}}= \frac{1.77}{3.83}=0.462. $$

**Figure 2:**
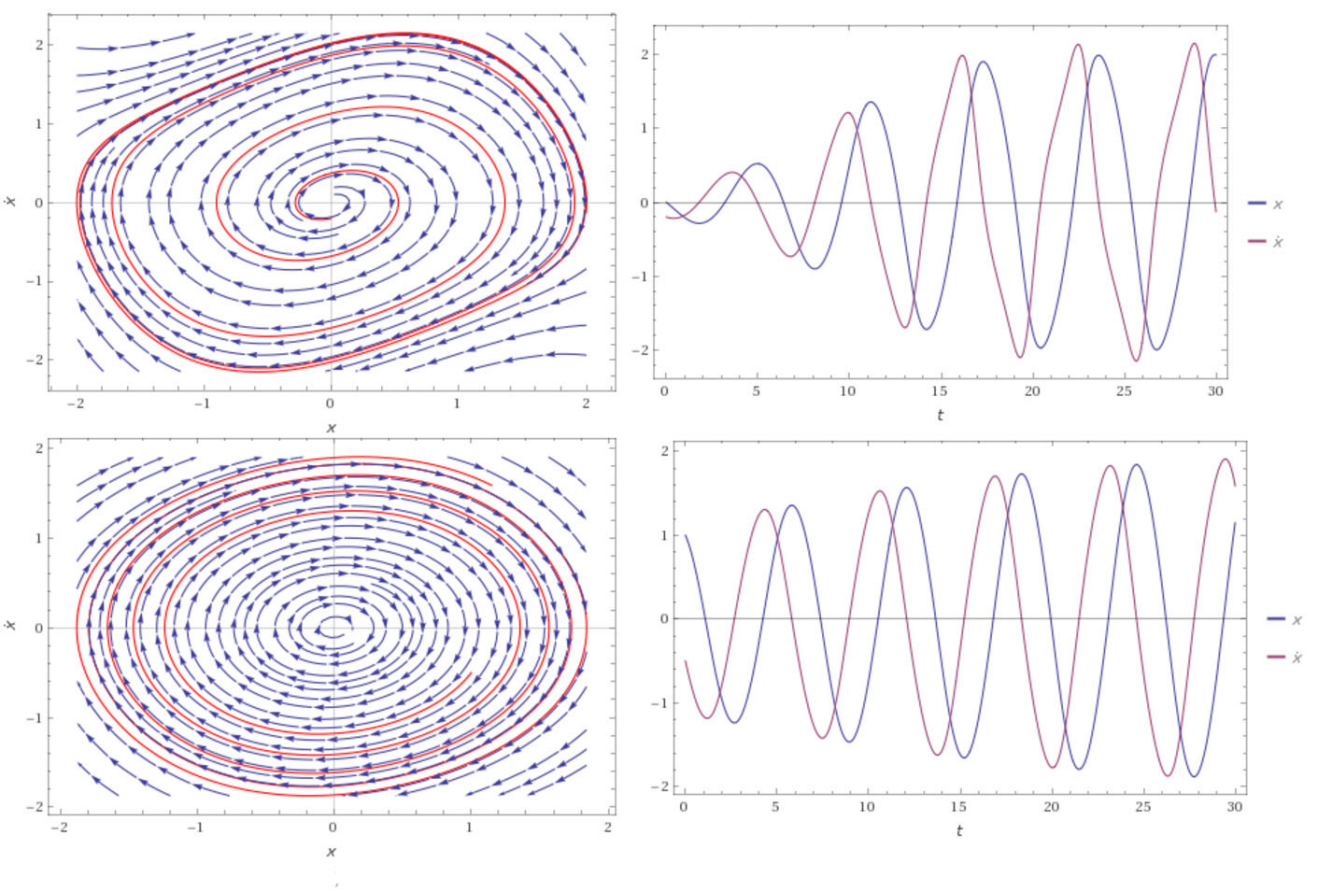
Solutions of (4) for different cases, depending on the value of *ρ*. We recognize that the cyclic solution for these cases is based on the fact that $\mathbb{Q}$ is cyclic

For instance, when $\rho =0.4$, we have an optimal solution with the initial condition $(\chi _{0},\Upsilon _{0})=(0,0)$. Furthermore, it is unstable cyclic because it indicates a portrait unstable limit cycle (see Fig. 2, the upper graphs). When $\rho =0.1$, the system admits an optimal solution with a portrait unstable limit cycle (see Fig. 2, the lower graphs).

#### Remark 3.1

The upper value of *ρ* plays an important role in describing the behavior of the optimal solution. It represents that when the upper value of *ρ* is equal to 0.33, then we have a stable solution (see Example 3.2). If the value is greater than 0.33, we have an unstable optimal solution (see Example 3.3). We shall take into account this fact when we establish the connections of Tutte polynomials in the next section.

### Approximated solution

In this section, we aim to use a type of polynomial to approximate the solution of (1). In this place, we suggest to use the Tutte polynomial because the formality of this polynomial involves two variables as follows (see [18]):

$$ T_{m}(\varsigma ,\tau )= \sum_{0\leq i \leq m, 0< j < m} \omega (i,j) \varsigma ^{i} \tau ^{j}. $$

By using the construction of Example 2.1, we have the fractional Tutte polynomial as follows:

$$\begin{aligned} T^{\mu }_{m}(\varsigma ,\tau ):={}& \jmath ^{\mu }T_{m}( \varsigma ,\tau ) \\ ={}& (1-\mu ) T_{m}(\varsigma ,\tau )+\frac{\mu }{\Gamma (\mu )} \int _{0}^{\varsigma } T_{m}(\tau ,\tau ) ( \varsigma -\tau )^{\mu -1}\,d\tau \\ ={}& (1-\mu ) \sum_{0\leq i \leq m, 0< j < m} \omega (i,j) \varsigma ^{i} \tau ^{j} \\ &{}+\frac{\mu }{\Gamma (\mu )} \int _{0}^{\varsigma } \biggl( \sum _{0\leq i \leq m, 0< j < m} \omega (i,j) \tau ^{i+j} \biggr) (\varsigma - \tau )^{\mu -1}\,d\tau \\ ={}& (1-\mu ) \sum_{0\leq i \leq m, 0< j < m} \omega (i,j) \varsigma ^{i} \tau ^{j} \\ &{}+\frac{\mu }{\Gamma (\mu )} \sum _{0\leq i \leq m, 0< j < m} \omega (i,j) \biggl( \int _{0}^{\varsigma }\tau ^{i+j} (\varsigma -\tau )^{\mu -1}\,d\tau \biggr) \\ ={}& (1-\mu ) \sum_{0\leq i \leq m, 0< j < m} \omega (i,j) \varsigma ^{i} \tau ^{j}+\sum_{0\leq i \leq m, 0< j < m} \omega (i,j) \bigl(\jmath ^{\mu }\varsigma ^{i+j} \bigr) \\ ={}& (1-\mu ) \sum_{0\leq i \leq m, 0< j < m} \omega (i,j) \varsigma ^{i} \tau ^{j} \\ &{}+\sum_{0\leq i \leq m, 0< j < m} \omega (i,j) \biggl( (1-\mu ) \varsigma ^{i+j}+ \frac{\mu \Gamma (i+j+1)}{\Gamma (i+j+1+ \mu )} \varsigma ^{i+j+\mu } \biggr) \\ ={}&\sum_{0\leq i \leq m, 0< j < m} \omega (i,j) \biggl((1- \mu )\varsigma ^{i} \tau ^{j}+ (1-\mu ) \varsigma ^{i+j}+ \frac{\mu \Gamma (i+j+1)}{\Gamma (i+j+1+ \mu )} \varsigma ^{i+j+\mu } \biggr). \end{aligned}$$

To determine the upper bound (approximated value) of $\omega (i,j)$, based on Theorem 2.2, we shall consider that these weights satisfy the upper bound of *ρ*, which is given by the formula

5 $$ \begin{aligned}\omega (i,j)& = \frac{\Gamma (\mu )}{\Gamma (\mu )+(1-\mu )+\bar{\epsilon }_{\mu }} \\ &= \frac{\Gamma (\mu )}{\Gamma (\mu )+(2-\mu )+ \frac{(\varsigma -\tau )^{\mu }}{\Gamma (\mu )}}. \end{aligned} $$

Note that $\lim_{\mu \rightarrow 1} \omega (\varsigma ,\tau )=0.33$ providing that $\varsigma -\tau =1$. This value is approximated with the upper bound of *ρ* in Example 3.2. By suggesting the solution of system (3) in terms of fractional Tutte polynomials, we have

$$ \chi (\varsigma )= \sum_{n=0}^{N} A_{n} (\varsigma ,\tau ) T^{\mu }_{n}( \varsigma , \tau ),\qquad \Upsilon (\varsigma )= \sum_{n=0}^{N} B_{n} ( \varsigma ,\tau ) T^{\mu }_{n}(\varsigma , \tau ). $$

**Figure 3:**
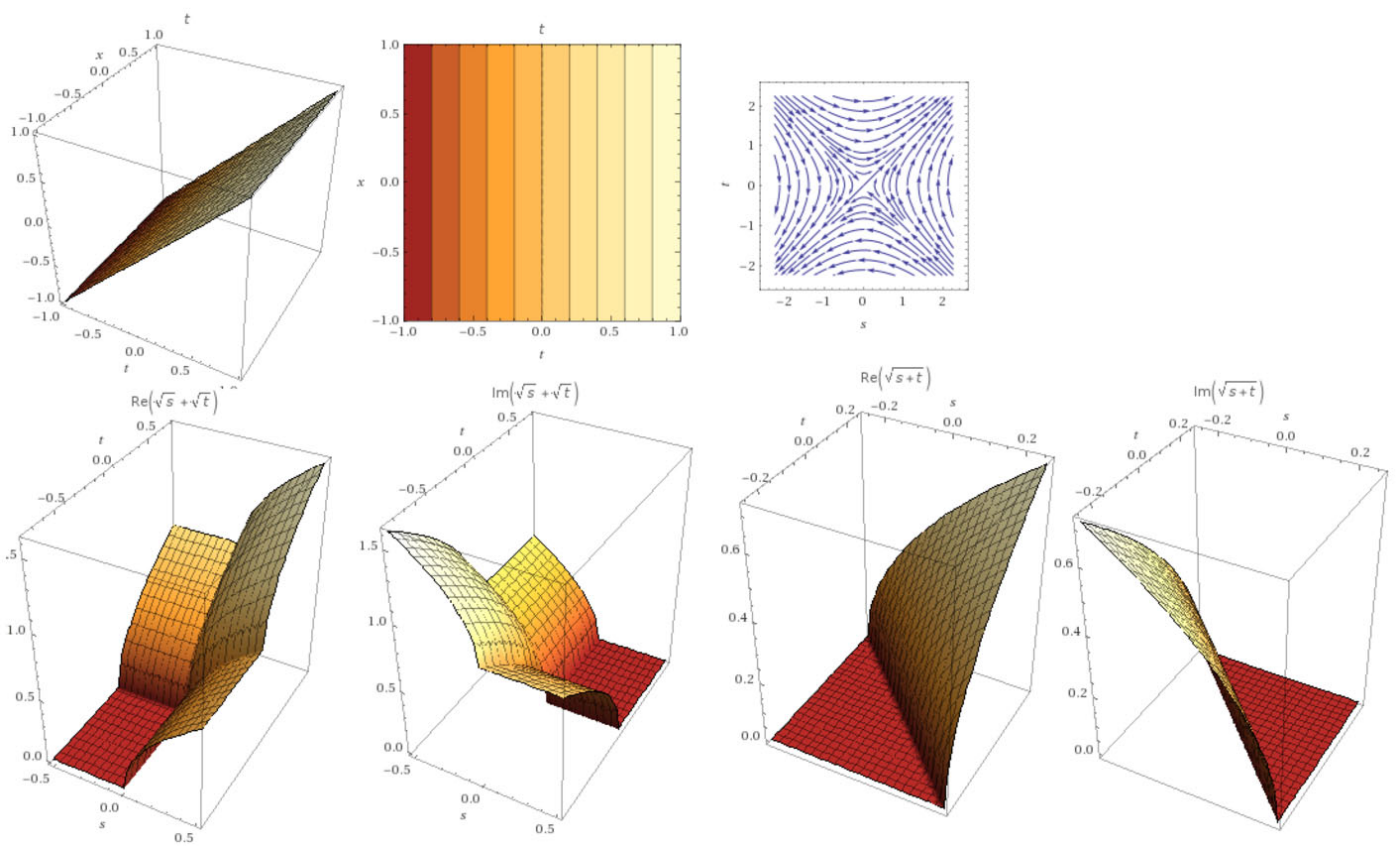
GB for the linear case. From the left: the graph represents 3D solution, contour plot, and integral curve. Similarly, for the case $(\tau ,\varsigma )$ for all real coefficients. The second row is GB of fractional order $\mu =0.5$, which indicates the set $\{\varsigma ,\tau ,\sqrt{\tau +\varsigma }, \sqrt{\varsigma }+\sqrt{ \tau }\}$

We suppose that *χ* and ϒ have the same gathering of roots as the original polynomials. That is, these polynomials can be recognized in a Grobner basis (GB). For linear functions in any number of variables, GB is analogous to Gaussian elimination. For example, if $(\chi (\varsigma ),\Upsilon (\varsigma ))= (\varsigma ,\tau )$, then $\operatorname{GB}(\varsigma ,\tau )=\{\varsigma ,\tau \}$ (see Fig. 3). For nonlinear cases, we have the following results.

#### Example 3.4

**Figure 4:**
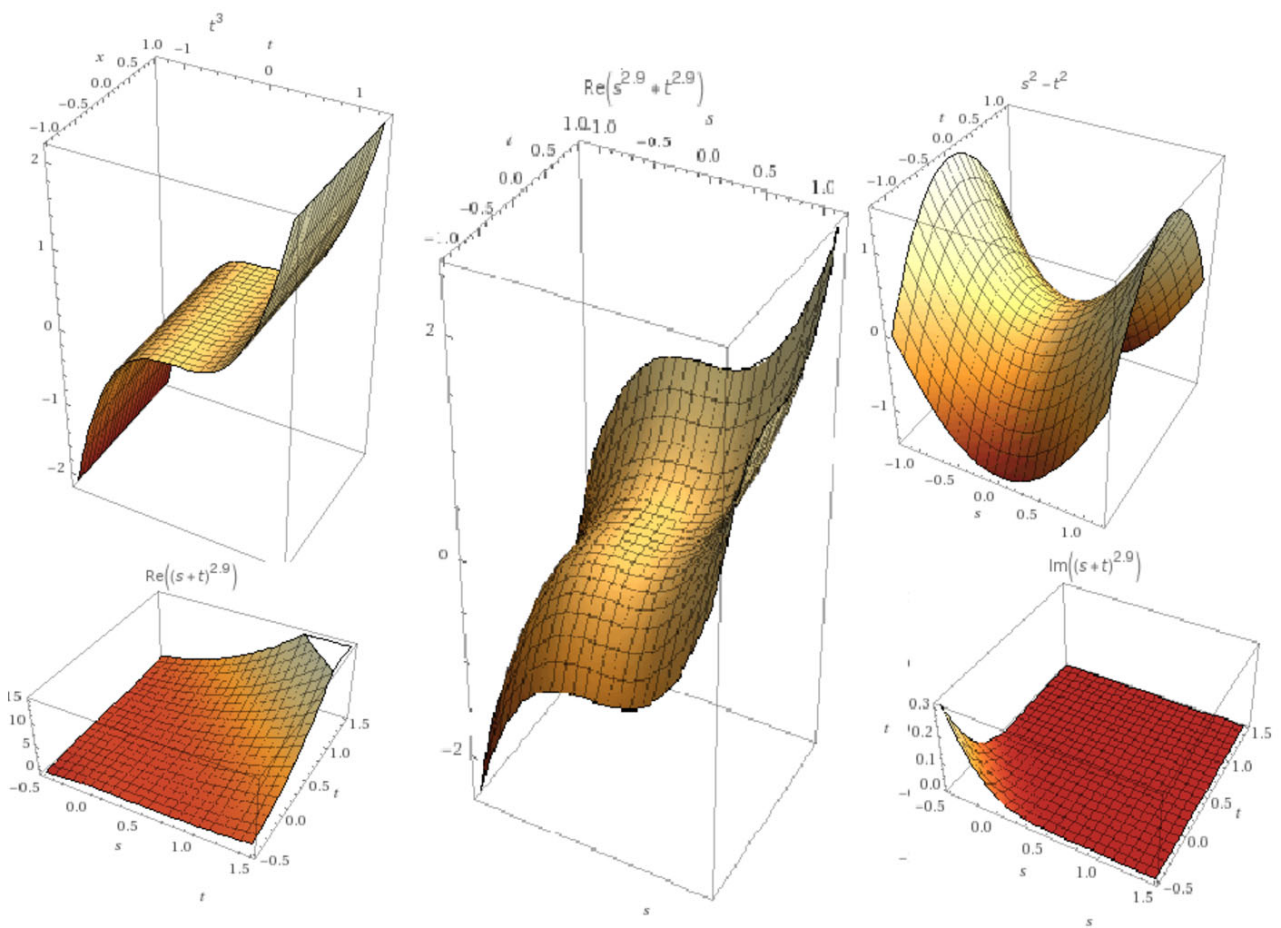
GB for the nonlinear case. From the left: the graph represents $\tau ^{3}$, *τς*, and $\varsigma ^{2}-\tau ^{2}$ respectively. The second row represents their fractional case when $\mu =0.9$

$\operatorname{GB}(\chi (\varsigma ),\Upsilon (\varsigma ))=\operatorname{GB} (\tau ^{2}-\varsigma ^{2}, \tau *\varsigma )=\{ \tau ^{3},\tau \varsigma ,\varsigma ^{2}-\tau ^{2} \}$ with $(0,0)$ root (see Fig. 4).

#### Example 3.5

**Figure 5:**
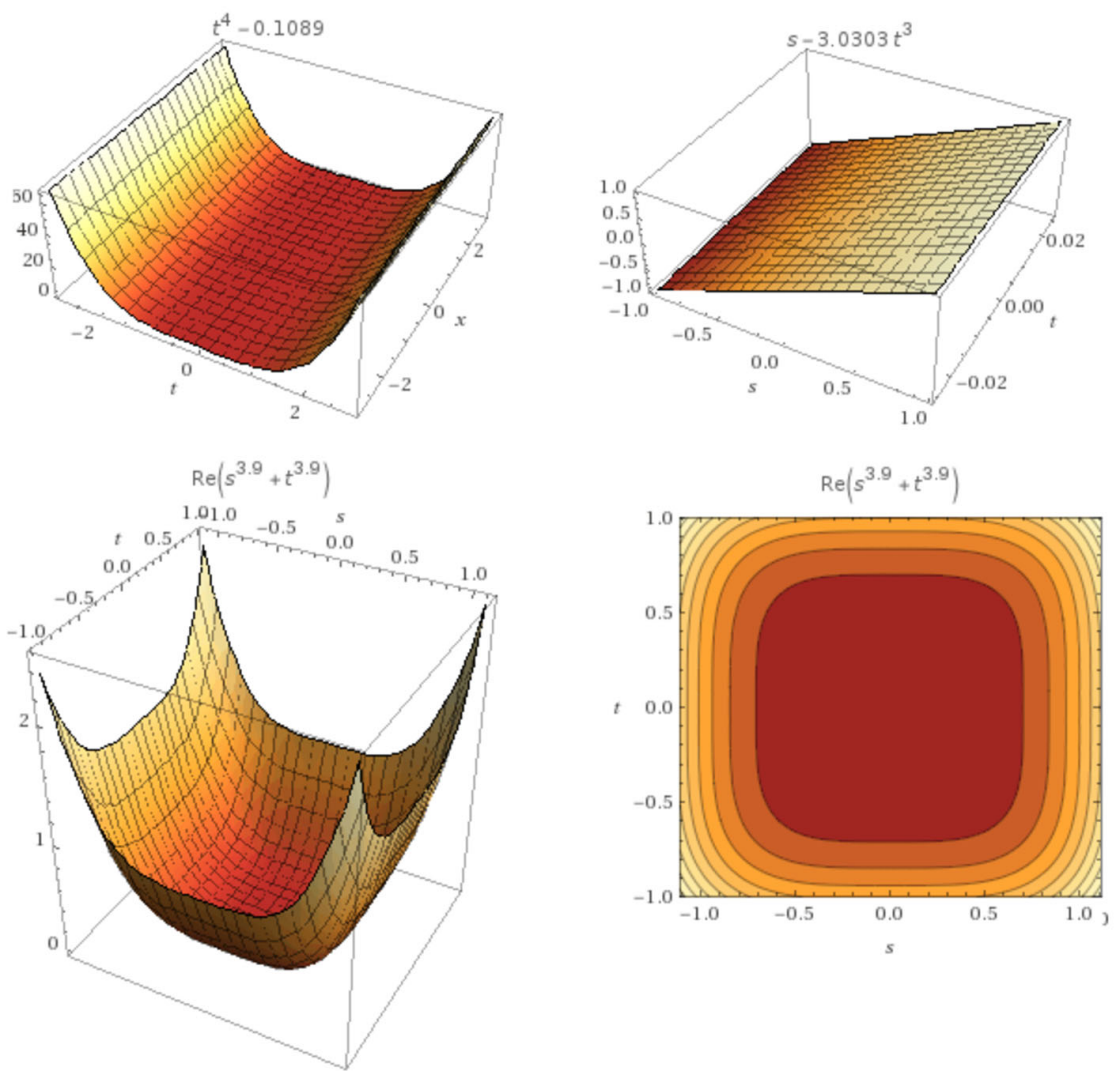
From the left: the graph represents $\tau ^{4}-0.1089$ and $\varsigma -3.03\tau ^{3}$ respectively. The second row represents the fractional case with $\mu =0.9$

$\operatorname{GB}(\chi (\varsigma ),\Upsilon (\varsigma ))=\operatorname{GB} (\tau ^{2}-\varsigma ^{2}, \tau *\varsigma -0.33)=\{ \tau ^{4}-0.1089,\varsigma -3.03\tau ^{3}\}$ with the real root $(\frac{\sqrt{33}}{10},\frac{\sqrt{33}}{10})$ (see Fig. 5).

#### Example 3.6

**Figure 6:**
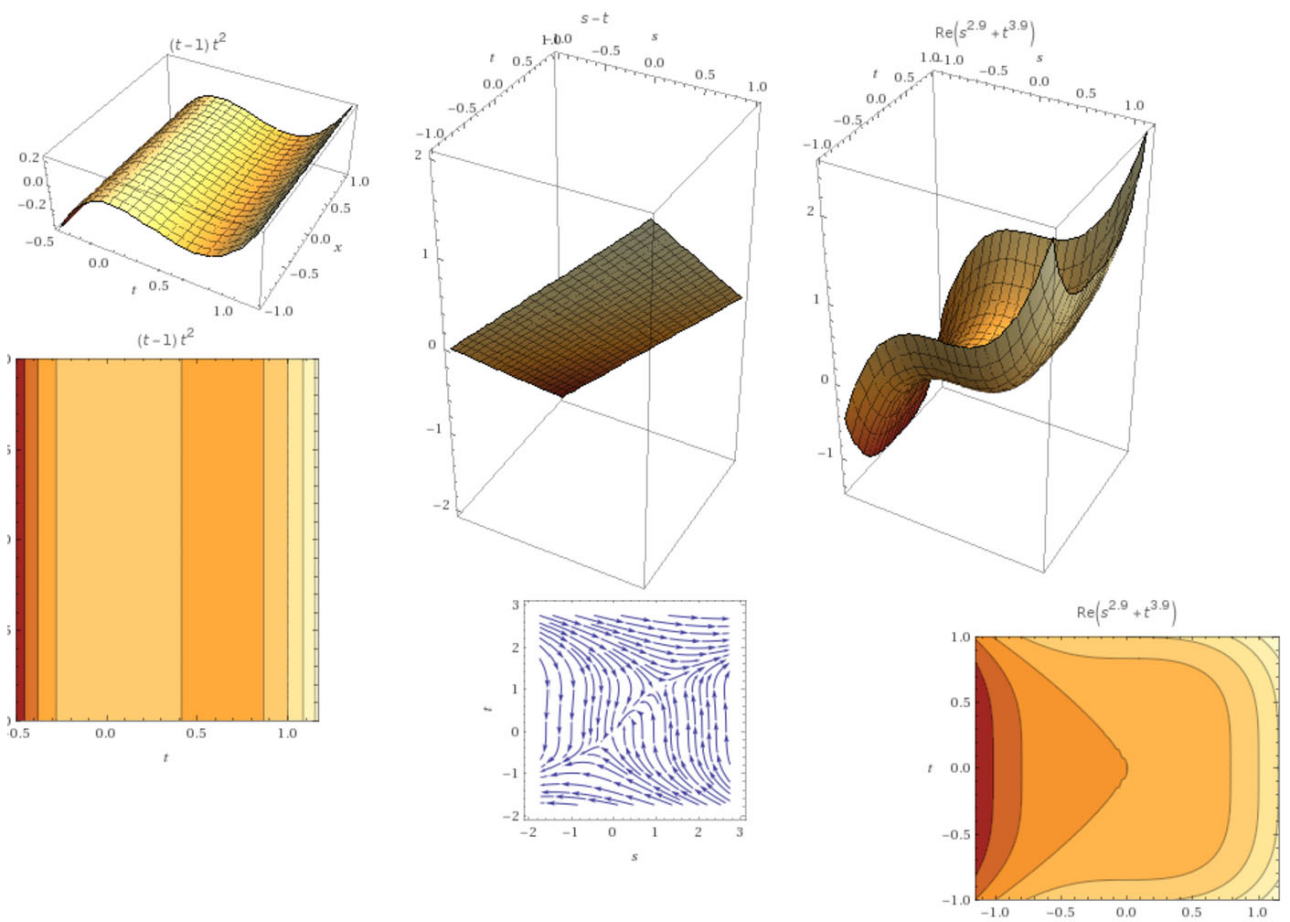
GB for the nonlinear case $\{ \tau ^{3}-\tau ^{2}, \varsigma -\tau \}$ with the fractional case when $\mu =0.9$

$\operatorname{GB}(\chi (\varsigma ),\Upsilon (\varsigma ))=\operatorname{GB} (\varsigma ^{3}-\tau ^{2}, \varsigma -\tau )=\{ \tau ^{3}-\tau ^{2}, \varsigma -\tau \}$ with two real roots $(0,0)$ and $(1,1)$ (see Fig. 6).

#### Example 3.7

**Figure 7:**
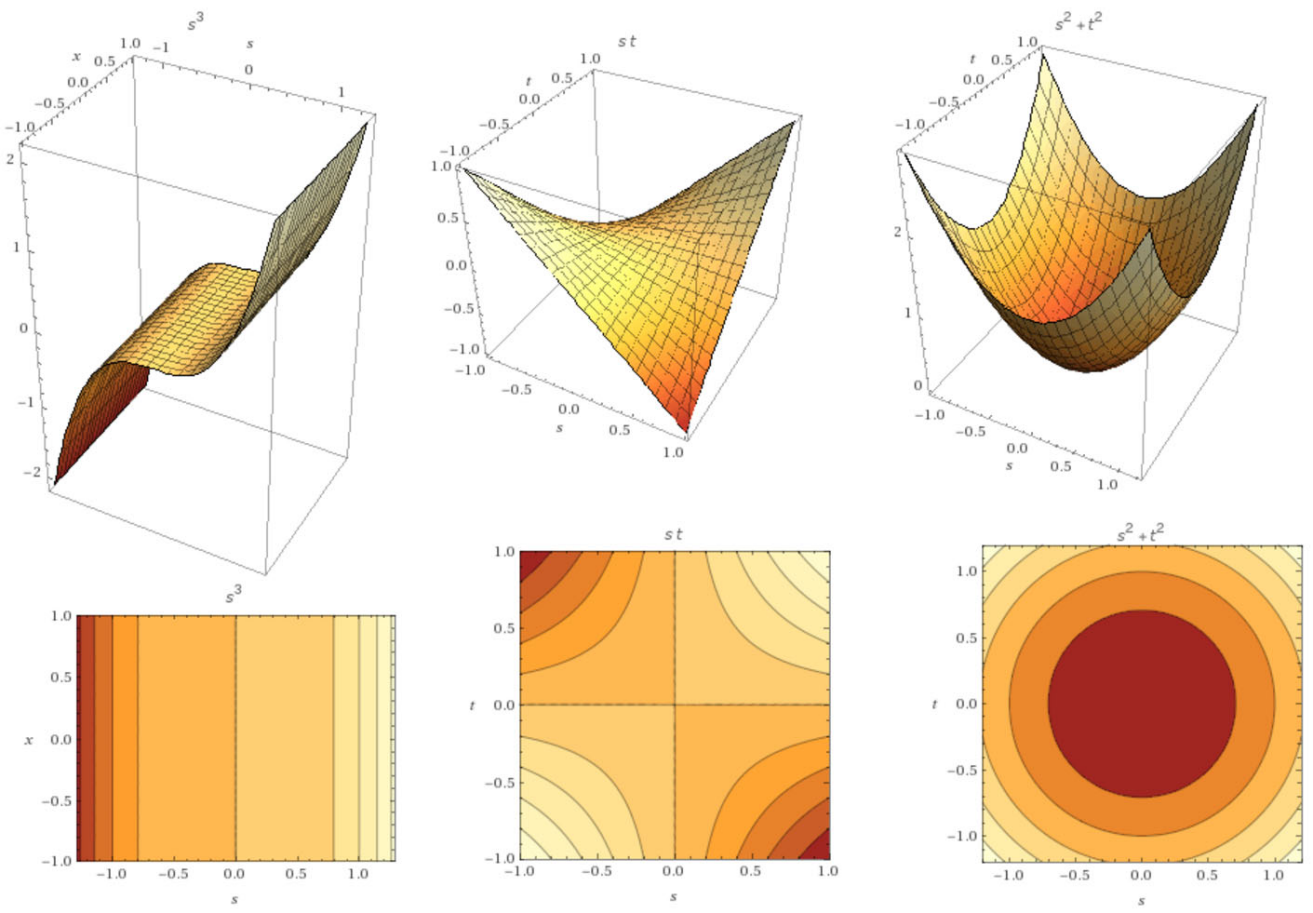
GB for the nonlinear case in Example 3.7

$\operatorname{GB}(\chi (\varsigma ),\Upsilon (\varsigma ))=\operatorname{GB} (\varsigma ^{3}+\tau ^{2}, \varsigma *\tau )=\{ \varsigma ^{3}, \varsigma \tau , \varsigma ^{2}+ \tau ^{2}\}$ with one real root $(0,0)$ (see Fig. 7).

### Application

**Figure 8:**
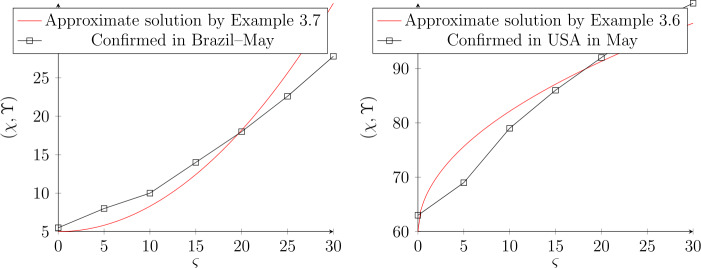
Approximate solution by using GB in May, where the data was given by $K=1000$. For Brazil, we select $\tau ^{2}=5$, while for USA is $\tau =60$. The fractional order is $\mu =0.95$ and $\mu =0.98$ respectively.

**Figure 9:**
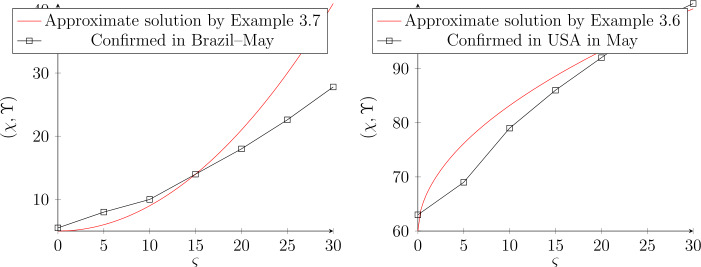
Approximate solution by using GB in May, where the data was given by $K=1000$. For Brazil, we select $\tau ^{2}=5$, while for USA is $\tau =60$. The fractional order is $\mu =1$ and $\mu =1$ respectively.

In this subsection, we shall utilize live data regarding COVID-19, which were recorded in May. Figures 8 and 9 indicate live data which were recognized in May for Brazil and USA. By using the approximated solution, we see that the data converge to the result of different BG values where the value of *τ* indicates the starting situation. Experimentally, we indicate that the good result appears, when the value of fractional order $\mu \rightarrow 1$ and the interval of convergence solution is $\mu \in (0.8,0.99] $.

## Conclusion

By using the fractional calculus, type ABC, we have generalized WCS. We have got two kinds of solutions, the first one is the optimal solution (see Theorem 3.1) using the concept of simulation function and the second is approximated solution using the GB set of polynomials of two variables $(\tau ,\varsigma )$. The optimal solution brings the stability, oscillation, and periodicity. The second solution is validated for discrete data. In this investigation, we generalized the most popular graph polynomials called the Tutte polynomial and a variety of carefully related graph polynomials such as the harmonic, movement, reliability, and shelling polynomials. We also used the Tutte polynomial to demonstrate how graph polynomials may be both dedicated and generalized, and how they can convert information relevant to medical applications. We concluded with a brief conversation of computational complexity deliberations. Different studies are presented using fractional calculus together with statistics and polynomials categories, which can be located in [19–25].

## Data Availability

Data sharing not applicable to this article as no data-sets were generated or analyzed during the current study.
